# Uncommon oral manifestation of lichen sclerosus: critical analysis of cases reported from 1957 to 2016

**DOI:** 10.4317/medoral.21606

**Published:** 2017-06-04

**Authors:** Saygo Tomo, Ingrid-da Silva Santos, Sâmia-Alves de Queiroz, Daniel-Galera Bernabé, Luciana-Estevam Simonato, Glauco-Issamu Miyahara

**Affiliations:** 1 Oral Oncology Center, São Paulo State University – UNESP, School of Dentistry, Araçatuba, Brazil; 2 University Brasil, School of Medicine, Fernandópolis, Brazil

## Abstract

**Background:**

Lichen sclerosus is a mucocutaneous autoimmune disease which might be initiated by infectious pathogens as Borrelia Bugrdorferi and HPV. This disease shows destructive potential and is rarely diagnosed in oral mucosa. The purpose of this paper is to evaluate the characteristics of cases described in literature from 1957 to 2016, looking to provide valuable evidence about clinicopathologic features of this disease.

**Material and Methods:**

A MedLine search was performed aiming to find oral lichen sclerosus cases in literature and discuss its demographical and pathological characteristics as well as treatment methods performed for these cases.

**Results:**

34 oral lichen sclerosus cases with histological confirmation and one clinicopathologic study linked with this disease were found in literature. Oral lichen sclerosus affected most commonly female patients, were asymptomatic and not associated to skin or genital lesions. Furthermore, affected patients in a range of 7 – 70-years old (Average age = 31.81).

**Conclusions:**

Oral lichen sclerosus is a rare pathologic process with slight predilection for prepubertal girls, for which topical corticosterois have demonstrated satisfactory therapeutic value.

** Key words:**Lichen sclerosus et atrophicous, skin diseases, mouth disease, autoimmune diseases, mouth.

## Introduction

In 1887 Hallopeau (1) described ‘lichen planus atrophicus’ as a new pathologic process occurring in anogenital region. Latter in 1892, Darier (2) published histopathologic features of a similar lesion named ‘lichen planus sclerosus’. Anyway, both lesions were considered variations of lichen planus (LP) by other researchers (3,4). Montgomery and Hill (5), in 1940, showed both lesions represented the same disease, with clinical and histopathologic features distinct from LP and described this disease as ‘lichen sclerosus et atrophicus’, which became a misused term along the years, once not all lichen sclerosus are atrophic (4).

Lichen sclerosus (LS) is now described as an unusual mucocutaneous chronic inflammatory disease which predominantly involves the anogenital region and demonstrates potential for resulting in atrophy, destructive scaring, functional impairment and malignant evolution (6). Therefore, early adequate diagnosis and either therapeutic intervention or clinical follow-up are mandatory (6). Although anogenital skin and mucosa is the mostly affected region, in 15% to 20% of cases (4,7,8) patients might be committed by extragenital lesions, and the oral mucosa is an extremely rare site for LS lesions occurrence (8-11), while only 6% of cases are represented by isolated extragenital lesions (6). Besides LS shows a slight predilection for prepubertal girls and pre and postmenopausal women, individuals at any age may be affected (6), as well as male individuals. To define any prevalence rate for LS lesions occurring in mouth is challenging, since, as previously mentioned, this anatomical site is rarely affected by LS lesions (4,11-14).

Although LS have been described as an autoimmune disease, factors which might initiate atypical immunologic response in skin and mucosas have not been well defined. Among factors which have been discussed as possible causes for LS, besides factors inherent to the patient, as individual diathesis and hormonal variations, environmental factors include trauma and an emerging infectious agent in Brazil associated to Lyme disease and morphea (Borrelia Burgdorferi) (6,11). Moreover, human papillomavirus (HPV) have been studied, and some authors suggests this virus might be responsible for a percentage of anogenital LS cases (15).

**Figure 1:**
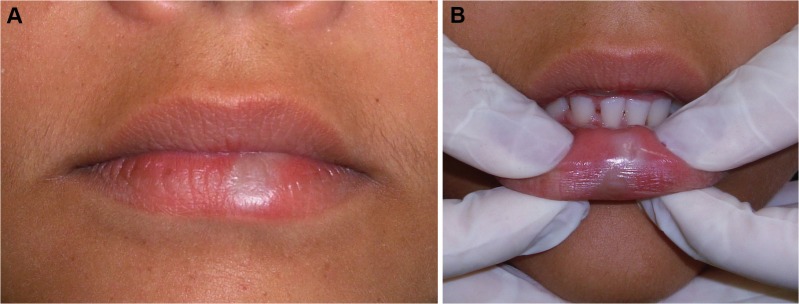
Clinical aspect of oral LS. (A) White rounded stain in lower lip vermilion with no surface alteration. (B) Extension of the lesion to lower lip mucosa.

Clinically, anogenital LS is typically characterized by well demarcated whitish plaques which might be slightly raised or flat le-sions and vary from small localized macula to extensive lesions involving larger areas of mucosa and skin (11). As shown in Figure 1, oral LS demonstrates these same clinical features described for anogenital LS. Patients with anogenital LS lesions usually relate soreness and pruritus, while these symptoms are less common in extragenital lesions (6). Diagnosis of LS is only obtained by histologic evaluation, since clinically, differential diagnosis for this disease includes whitish plaque lesions, as well as LP and vitiligo (6,16). Histopathologic features typically found for LS lesions include either variable epithelial atrophy or hyperplasia, focal hydropic degeneration of basal cells, a band of subepithelial hyalinization and a slightly diffuse band-like lymphocytic infiltrate beneath hyalinized collagen area (11,12). Both lichen planus and vitiligo lack the subepithelial hyalinized area, and furthermore, melanocytes presence excludes vitiligo hypothesis (6).

Regarding treatment, LS is a chronic disease with no cure and no available either systemic or local effective therapy; although, treatment can relive symptoms and improve aesthetic aspects. Oral lesions have been successfully treated either by topical corticosteroids administration or surgical excision (4,13,17,18).

Given that LS is greatly rare in oral mucosa, to perform researches aiming to describe demographic and clinicopathologic characteristics of this disease with oral manifestation becomes challenging, therefore, in this paper we critically analyzed the few cases described in literature aiming to provide scientific valuable data regarding LS of the oral mucosa.

## Material and Methods

This study was performed through search in MedLine database searching for papers reporting oral LS cases with histological diagnosis confirmation from 1957 to 2016 on English literature ( Table 1). To analyze these cases, we extracted from each paper data regarding age, sex, symptomatic manifestation of the lesion, presence of skin lesions and presence of anogenital lesions. As the characteristics that we aimed to evaluate were not available for all cases, percentages are presented based on the total of cases which had each variable information available.

**Table 1:**
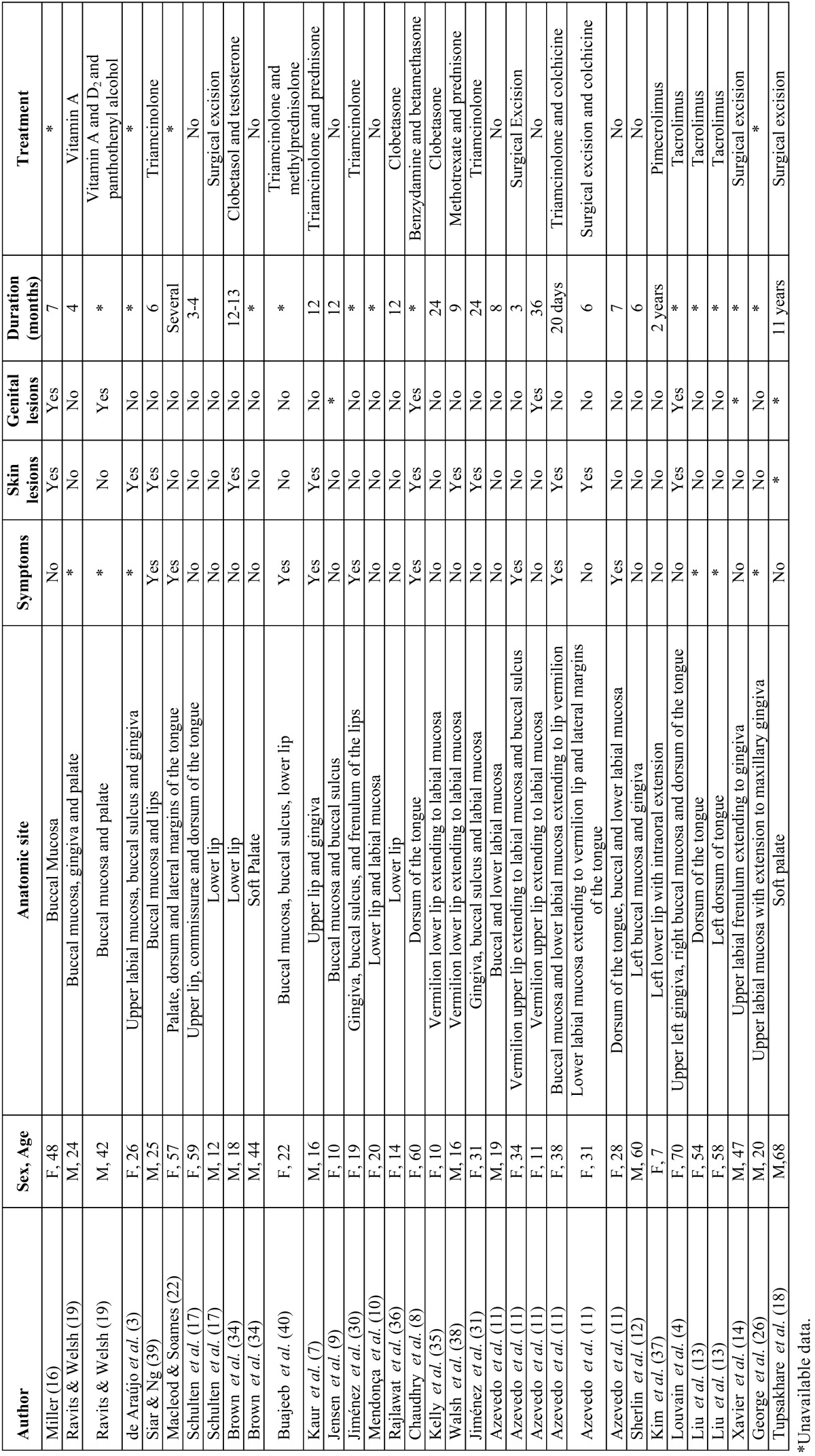
Sumary of oral lichen sclerosus reported from 1957 to 2016.

## Results

In our search, we found 34 cases of oral LS with histopathologic confirmation of the diagnosis ( Table 1). Analysis of these cases is summarized in  Table 2. Our critical analysis showed high age variability, ranging from 7 to 70 years old, while the average age was 31.81. Females individuals were clearly more affected (61.73%) by oral LS than male individuals (38.23%), and in most of cases, symptomatic manifestation of the lesions was not present. Furthermore, in only 33.33% of the cases, oral lesions were associated to skin lesions, and in only 16.12% of the cases oral LS lesions were associated to genital lesions. Duration of the lesions varied from 20 days to 2 years and the most commonly affected site was the lip (vermilion and mucosa).

**Table 2:**
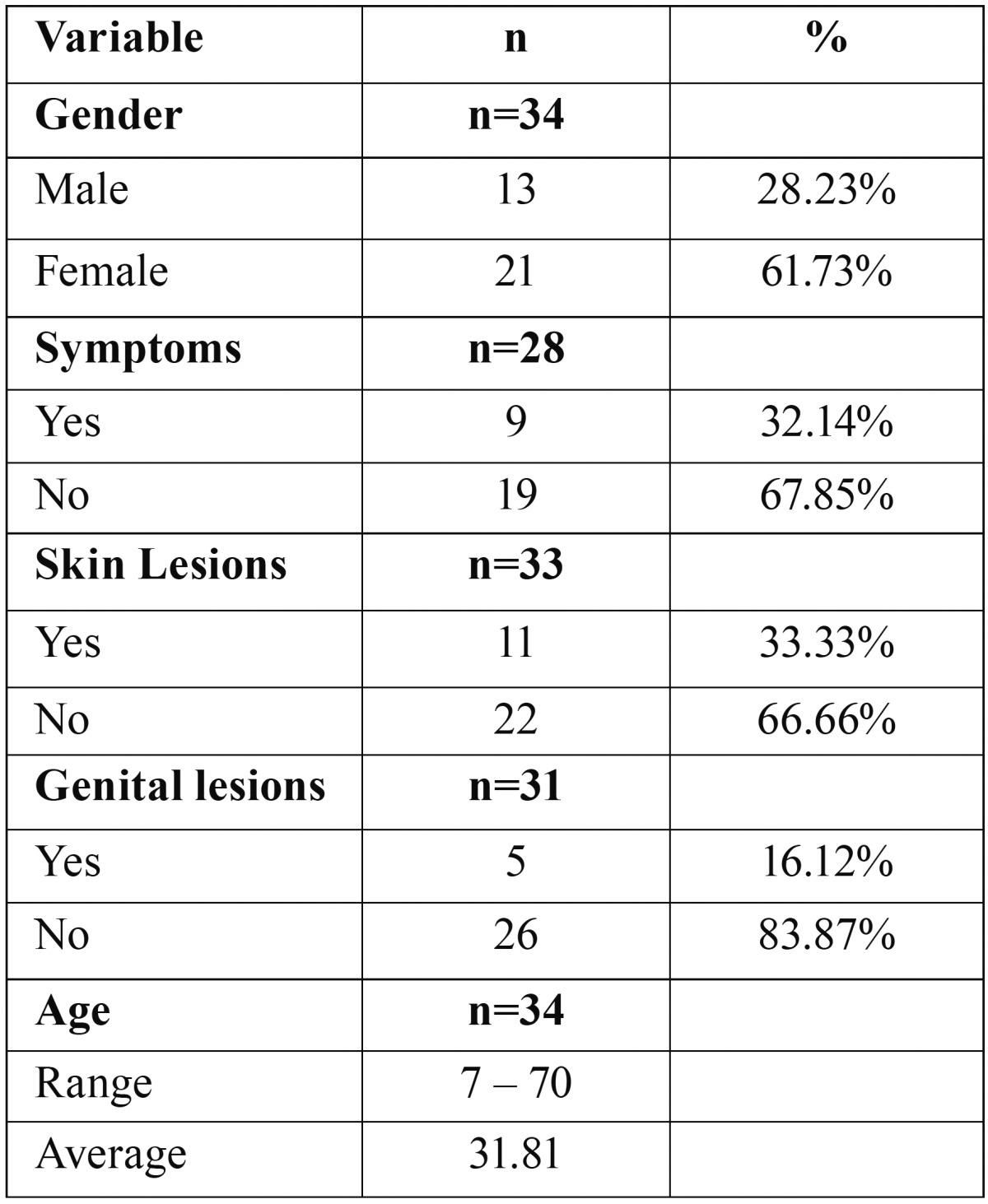
Characteristics of oral lichen sclerosus cases reported from 1957 to 2016.

## Discussion

The term lichen sclerosus (LS) describes a chronically relapsing dermatosis with potential to result in destructive scaring, atrophy, functional impairment, and malignant transformation, which predominantly affects anogenital mucosa and skin (6,11,16). As demonstrated in our review, oral manifestation of LS is an extremely rare condition, with 34 histologically confirmed cases reported since 1957, when Miller (16) and Ravits and Welsh (19) published the first papers describing oral LS cases (Table 1).

In review performed by Fistarol & Itin (6), the authors reported anogenital LS is clearly more frequent among female patients compared to male patients and shows a remarkable predilection for prepubertal girls and post-menopausal women. Recently, Knio *et al.* (20) demonstrated in clinicopathologic study that among 60 patients with LS (genital and extragenital) 70% were women. Attili and Attili (21) did not found predilection for any gender in a clinicopathologic study of lips LS, however, we observed that oral LS demonstrated a slight predilection for the female gender ( Table 2). In addition, we also noted that oral LS shows high age variability, occurring in patients from 7 to 70-years old ( Table 2). Although LS occurrence in the anogenital mucosa and skin have been reported as more frequent in young girls and post-menopausal women, we found no predilection for any age in our review.

Clinical features of skin and anogenital LS include white stains which might be slightly elevated or flat, with well demarcated limits and variable shape (6). In our 34 reported cases review the lesions’ characteristics described, specially size, color and shape, do not differ from skin and anogenital LS characteristics. However, further diseases, as well as LP, vitiligo and oral leukoplakia (OL) must be considered on the differential diagnosis of LS. Macleod and Soames (22) reported a case of oral LS in comorbity with vitiligo. Therefore, histopathologic analysis of biopsy specimens is required for obtaining the diagnosis of LS (6,11). In general, histopathologic characteristics of oral LS are not highly different from the characteristics of skin and anogenital LS. Knio *et al.* (20) indicated genital LS lesions were more likely to present epithelial hyperplasia, while extragenital lesions were mostly associated to epithelial atrophy. However, the main histologic feature of LS is the band of hyalinized collagen fibers immediately below the epithelium, which might show variable density, organization and thickness. Furthermore, a band-like inflammatory infiltrate is found below the hyalinized area. It is suggested that the inflammation begins near to the epithelium, leading to destruction and reorganization of collagen fibers and leaving conjunctive sclerosis as it deepens into the tissue. Thus, older lesions of LS will present thicker band of hyalinized collagen while in lesions with short period of evolution this hyalinization band are thinner (6).

Literature shows in only about 6% of LS cases, lesions occurs in extragenital isolated sites (6). Oral LS most commonly occurred isolate, since in only 11 (33.33%) cases oral LS occurred associated to skin lesions and in only 5 cases (16.12%) oral LS was associated to genital lesions ( Table 2), suggesting skin lesions are more commonly associated to genital lesions, while oral lesions tend to occur isolate.

The etiology of LS was not well defined, but there is a general agreement regarding the auto-immune nature of this disease (6,11); more specifically, anogenital lesions have been associated to extracellular matrix 1 (ECM1) protein destructions, which may be mediated by circulating IgG antibodies and, furthermore, might also be associated to ECM1 autoreactivity (6,14). ECM1 protein develops an important role on the control of keratinocyte differentiation within epidermis, whereas within dermis ECM1 is important for structural organization (23). Xavier *et al.* (14) described an oral LS case in which molecular tests suggested extracellular matrix reorganization, elastic fibers reduction and altered proteolytic activity, however, the cause of this reorganization was not investigated. Moreover, anogenital LS has also been associated to patient diathesis, and triggering factors which may lead to LS occurrence include trauma and chronic irritation, hormonal influences and infectious pathogens such as Borrelia Burgdorferi, EBV and HPV, leading some authors to describe LS as a multifactorial disease (6,11,24,25).

In a case reported by George *et al.* (26), Borrelia Burgdorferi was identified by Focus-Floating microscopy. Borrelia Burgdorferi is the cause of the Lyme Borreliosis, which has the LS as the most common skin condition associated (27), deserving attention from the health care professionals. Moreover, it was demonstrated Borrelia Burgdorferi has the potential to induce collagen and elastic fibers alterations, which is the main histopathologic finding of LS lesions (27).

The association of HPV to anogenital LS have been described in variable percentage rates by several authors. In 1991, Kiene *et al.* (28), by polymerase chain reaction (PCR) technique of paraffin embedded biopsy specimens of LS of the vulva observed that HPV-16 was present in 4 out of 18 samples, however this study only evaluated the presence of HPV-16 while further genotypes of the virus could be present in the specimens. Nasca *et al.* (25) indicated 17.4% of 46 patients with penile LS were positive to HPV infection by brush cytology of the lesions. Prowse *et al.* (29), found a higher HPV prevalence of 33% among 20 patients with penile LS. HPV presence was not investigated in any of the 34 cases of oral LS included in our review, demonstrating that there is no available data regarding the association of HPV and oral LS, suggesting the need for further investigations in future cases and studies.

Most of cases found in literature had the lips committed by LS ( Table 1), but factors which might initiate an inflammatory response leading to this autoimmune reaction in this site have not been discussed. We believe this might be explained by the fact that the lips are the mouth sites most susceptible to linear environmental stimuli, as infectious pathogens and trauma (Köbner phenomenon), however, the etiology of oral LS is still subject for investigation.

Anogenital LS is known to possibly result in loss of tissue function and structure when diagnosed and treated in advanced stages (6,11). Jiménez *et al.* (30), described an oral LS case with upper lip, buccal sulcus and gingiva involvement which in the lesion resulted in severe destruction of periodontal structures, with loss of bone tissue and dental mobility. Later, in 2008, the same team described another case of oral LS with severe gingival recession (31). These cases highlight oral LS lesions may present the destructive behavior reported for anogenital lesions, therefore, early diagnosing and treating these lesions might prevent significant structural losses and functional impairment.

Transformation of anogenital LS into squamous cell carcinoma (SCC) is subject of debate in literature. Although data regarding this transformation remains highly inconsistent, increased risk for anogenital SCC development in patients with anogenital LS is evident. Malignant transformation of vulvar LS rates varies from 1.4% to 61% and from 4% to 8% for penile LS, however, 32% - 50% of SCC of the penis demonstrate histologic findings suggestive of LS (15). Nevertheless, factors associated to malignant transformation of anogenital LS are not well elucidated. HPV infection was associated to variable percentages of genital LS which transformed into SCC, although, the presence of vulvar intraepithelial neoplasia (VIN) in vulvar LS seems to be more strongly associated to this transformation and the presence of HPV in this process might be a chance occurrence event per some authors (15). In 2011, Regauer (32) demonstrated that the persistence of LS lesions after surgical removal of anogenital SCC was associated to a recurrence rate of 50%. Moreover, Bleeker *et al.* (33), indicated a concurrent association between advanced age (>70 years-old) and VIN in the malignant transformation of LS of the vulva. Thus, the potential of anogenital LS to transform into SCC requires further investigation, including the role of the HPV in this process. In our review, no cases of oral LS were reported with transformation into SCC until this moment, which anyway does not exclude the need for early diagnosing and treating LS in mouth.

Treatment approaches for LS generally aims to release symptoms, to prevent tissue destruction and to improve cosmetic aspects, given that there is no available effective therapy and cure for this disease (1,6). Although anogenital LS lesions are commonly symptomatic, presenting soreness and pruritus, most of oral LS cases described were asymptomatic (67.85%), which might represent a worrisome fact for the clinician, since the asymptomatic development of a disease does not aware the patient to early look for professional care. Anogenital lesions of LS used to be treated with testosterone applications; nevertheless, this approach became misused due to lacks in effectiveness and hormonal instabilities in female patients (6). Surgical excision is reliable for small oral and vulvar LS lesions (11). We found that 4 out of 34 oral LS described cases were surgically excised ( Table 1), leading to symptoms reduction and no recurrence (11,14,18). Local administration of corticosteroids has been reported with high success rates for anogenital lesions (6), whereas oral LS cases reported in literature were mostly treated by local administration of corticosteroids with satisfactory success (8,34-38). Triamcinolone have shown good results for the treatment of anogenital LS (6), and several authors who reported oral LS cases in literature described local administration of triamcinolone as treatment with satisfactory symptoms reduction and esthetical aspects improvements (7,11,30,31,39,40).

## Conclusions

LS is an uncommon and distinct autoimmune condition with rare oral manifestation, therefore, scientific evidence regarding sociodemographic and clinicopathologic characteristics of oral LS lesions are difficult to establish. Nevertheless, data extracted from few cases found in literature allow to elucidate that some characteristics of these lesions might corroborate with characteristics described for skin and anogenital lesions regarding etiologic factors, pathogenesis and therapeutic approaches.
